# Protocol for differentiation and efficient AAV-mediated gene delivery to hiPSC-derived microglia for functional studies

**DOI:** 10.1016/j.xpro.2026.104455

**Published:** 2026-03-30

**Authors:** Christina N. Heiss, Anastasia Mihai, Stefanie Fruhwürth, Andreas Björefeldt

**Affiliations:** 1 Department of Psychiatry and Neurochemistry, Institute of Neuroscience and Physiology, University of Gothenburg, Gothenburg, Sweden; 2 Department of Physiology, Institute of Neuroscience and Physiology, University of Gothenburg, Gothenburg, Sweden

**Keywords:** Neuroscience, Stem Cells, Tissue Engineering

## Abstract

Human induced pluripotent stem cell (hiPSC)-derived microglia (hiMG) constitute a powerful platform to study human microglial biology in health and disease. Here, we present a protocol for microglial differentiation and efficient gene delivery to these cells using adeno-associated virus (AAV). We describe steps for differentiation, viral transduction, and validation using flow cytometry and fluorescence imaging. Finally, to demonstrate the utility of this approach, we outline procedures to record and analyze calcium activity in hiMG using the genetically encoded sensor GCaMP8s.

For complete details on the use and execution of this protocol, please refer to Fruhwürth et al.^1^ and Heiss et al.^2^

## Highlights

• Steps for differentiation of human iPSC-derived microglia (hiMG)
• Instructions for efficient gene delivery to hiMG using adeno-associated virus
• Guidance on how to perform calcium imaging in hiMG using a genetically encoded sensor

## Abstract (editor highlights)

Publisher’s note: Undertaking any experimental protocol requires adherence to local institutional guidelines for laboratory safety and ethics.

## Graphical abstract

**Figure:**
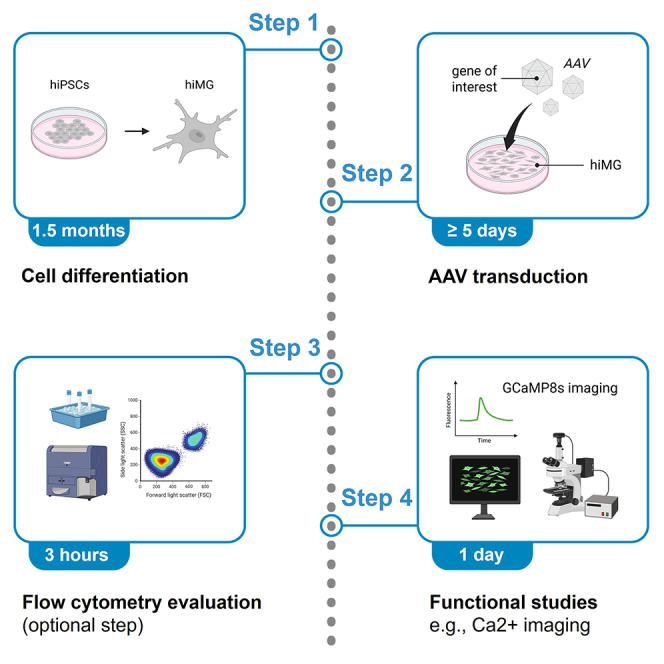

## Teaser

Human induced pluripotent stem cell (hiPSC)-derived microglia (hiMG) constitute a powerful platform to study human microglial biology in health and disease. Here, we present a protocol for microglial differentiation and for delivering genes to these cells using adeno-associated virus (AAV). We describe steps for differentiation, viral transduction, and validation using flow cytometry and fluorescence imaging. Finally, to demonstrate the utility of this approach, we outline procedures to record and analyze calcium activity in hiMG using the genetically encoded sensor GCaMP8s.

## Before you begin

Microglia are central nervous system (CNS)-specialized parenchymal macrophages with key roles in brain homeostasis, innate immunity, and neurodegenerative pathology.^3^^,^^4^ hiMG has become a key *in vitro* platform for disease modelling and basic research. The differentiation protocol described here is based on a previously published serum- and feeder-free protocol^5^^,^^6^ which yields hiMG with substantial transcriptomic overlap with primary human microglia.^6^ This protocol recapitulates the embryonic development of microglia by induction of hematopoiesis and myeloid differentiation. Combined with the use of genetic tools such as CRISPR-assisted editing and screening, as well as genetically encoded actuators and sensors, this cell model holds great promise for uncovering novel disease mechanisms and providing new insights into microglial function.

Importantly, efficient and non-toxic systems for gene delivery are crucial to support robust evaluation of manipulations at DNA, RNA and protein level in hiMG. As a non-integrating vector, adeno-associated virus (AAV) is known for high safety, low immunogenicity, and long-term gene expression in non-dividing cells.^7^ This protocol provides instructions for efficient viral transduction of hiMG using a recently developed AAV capsid variant.^8^ While used here to express a genetically encoded fluorescent reporter and calcium sensor, the approach should support delivery of diverse payloads to hiMG for use in a broad range of basic research and therapeutic applications.

Intracellular calcium signaling in microglia is triggered by activation of ionotropic and metabotropic membrane receptors (e.g., purinergic, glutamatergic) to facilitate key sensor and effector functions, and this process may become dysregulated in aged and diseased brains.^9^ Genetically encoded indicators can offer insights into the functional role of calcium signaling in these cells across multiple contexts (e.g., disease and sex-specific) and serve as a tool to couple morphological and transcriptomic profiles to physiological roles. Here we provide a protocol to express and validate GCaMP8s for calcium imaging in hiMG using artificial cerebrospinal fluid (ACSF) perfusion and chemical stimulation with ATP.

### Innovation

In a recent study we identified the cMG capsid^8^ (originally described to mediate efficient *in vitro* transduction of primary mouse microglia) as a high-efficacy/low-toxicity gene delivery tool for hiMG.^2^ Here we provide an optimized protocol for transducing hiMG using this AAV-based approach together with instructions for quantitative validation and application in calcium imaging. All together, we present a streamlined and validated protocol to differentiate and efficiently transduce hiMG for functional studies.

Before beginning the protocol, ensure that all necessary requirements (e.g., institutional permissions, laboratory facilities, training) for work with human iPSC lines and viral vectors are met.

## Key resources table

**Table:** 

REAGENT or RESOURCE	SOURCE	IDENTIFIER
**Antibodies**		

IBA1 monoclonal antibody	Synaptic Systems	234017
TMEM119 polyclonal antibody	Proteintech	27585-1-AP
GPR34 polyclonal antibody	Invitrogen	PA5-99041
CD68 monoclonal antibody	Bioscience	14-0688-82
Donkey anti-rat IgG Alexa Fluor 488	Thermo Fisher Scientific	A-21208
Donkey anti-rabbit IgG Alexa Fluor 488	Thermo Fisher Scientific	A-21206
Donkey anti-mouse IgG Alexa Fluor 488	Thermo Fisher Scientific	A-21202

**Bacterial and virus strains**		

AAV-cMG	Lin et al., 2022^8^	N/A

**Chemicals, peptides, and recombinant proteins**		

2-mercaptoethanol	Thermo Fisher Scientific	31350010
Anti-adherence rinsing solution	Stemcell Technologies	07010
DMEM	Thermo Fisher Scientific	31966021
DMEM/F12 GlutaMax x1	Thermo Fisher Scientific	31331–028
Dulbecco’s phosphate buffered saline without Ca^2+^ and Mg^2+^, DPBS (−/−)	Thermo Fisher Scientific	14190–086
GlutaMax	Thermo Fisher Scientific	35050038
Human recombinant BMP-4, 10 μg	Peprotech	120-05ET
Human recombinant GM-CSF, 20 μg	Peprotech	300–03
Human recombinant IL-3, 10 μg	Peprotech	200–03
Human recombinant IL-34, 10 μg	Peprotech	200–34
Human recombinant M-CSF, 10 μg	Peprotech	300–25
Human recombinant SCF, 10 μg	Peprotech	300–07
Human recombinant VEGF-121, 10 μg	Peprotech	100-20A
Matrigel	Thermo Fisher Scientific	11573560
mTesr plus media 500 mL	Stem cell technologies	100–0276
Penicillin Streptomycin sol. HyClone/GE	VWR	SV30010
TrypLE Express (1×)	Thermo Fisher Scientific	12604013
UltraPure EDTA (0.5 M, pH 8.0)	Thermo Fisher Scientific	15575–020
X-VIVO 15, 500 mL	VWR (Lonza)	LONZBE02-060F
Y-27632 dihydrochloride (Rock inhibitor), 1 mg	Peprotech	1293823
Histofix	Histolab	1000
TBS-Tablets	Medicago AB	09-7500-10
Donkey serum	Sigma Aldrich	D9663
Triton X-100	Sigma Aldrich	HFH10
DAPI (4′,6-Diaminidino-2-Phenylindole, Dihydrochloride)	Thermo Fisher Scientific	D1306
ProLong Gold antifade reagent	Thermo Fisher Scientific	P36930
LIVE/DEAD™ Fixable Far Red dye	Thermo Fisher Scientific	L34973
NucBlue™ dye	Thermo Fisher Scientific	R37605
Trypsin-EDTA, 0.25%	Thermo Fisher Scientific	25200056
Fetal bovine serum	Thermo Fisher Scientific	10082147
Phosphate-buffered saline (PBS), pH 7.4	Thermo Fisher Scientific	10010023
Doxorubicin hydrochloride	Thermo Fisher Scientific	J64000-MA
Sodium chloride (NaCl)	Sigma-Aldrich	S2014; CAS: 7647-14-5
Sodium dihydrogen phosphate (NaH_2_PO_4_)	Sigma-Aldrich	
Sodium bicarbonate (NaHCO_3_)	Sigma-Aldrich	S5761; CAS: 144-55-8
Potassium chloride (KCl)	Sigma-Aldrich	P9333; CAS: 7447-40-7
D-glucose	Sigma-Aldrich	G8270; CAS: 50-99-7
Calcium chloride hexahydrate (CaCl_2_)	Sigma-Aldrich	C3881; CAS: 10035-04-8
Magnesium chloride hexahydrate (MgCl_2_)	Sigma-Aldrich	M2670; CAS: 7791-18-6
Adenosine 5′-triphosphate disodium salt hydrate	Sigma-Aldrich	A6419; CAS:34369-07-8
Dimethyl sulfoxide (DMSO)	Sigma-Aldrich	D2650; CAS: 67-68-5

**Experimental models: Cell lines**		

Human induced pluripotent stem cell line	EBiSC/Sigma-Aldrich	WTSli051-A

**Recombinant DNA**		

rAAV2/cMG Rep/Cap plasmid	Addgene	#184539
AAV helper plasmid	Addgene	#112867
pAAV-SSFV-mScarlet expression plasmid	Heiss *et* al., 2025	N/A
pAAV-SSFV-GCaMP8s expression plasmid	This paper	N/A

**Software and algorithms**		

NIS-Elements (version 5.42.02)	Nikon Instruments	https://www.microscope.healthcare.nikon.com/products/software/nis-elements; RRID: SCR_014329
Fiji (ImageJ2 version 2.16.0/1.54p)	Schindelin et al.^10^	https://fiji.sc; RRID: SCR_002285
Visual Studio Code (version 1.100.2)	Microsoft	https://code.visualstudio.com; RRID: SCR_026031
FACSDiva™	BD Biosciences	N/A
FlowJo™	BD Biosciences	N/A
GraphPad Prism	Dotmatics	N/A

**Other**		

Cell strainer 50 μm, BD Falcon	Sigma Aldrich	CLS352350
AggreWell™ 800, 24-well plate	Stemcell Technologies	34811
Cell counter	ChemoMetec	NC-200
6-well plates, Primaria	Corning	353846
24-well plates	Thermo Fisher Scientific	144530
96-well plates	Thermo Fisher Scientific	260860
Centrifuge Labofuge 400	Thermo Fisher Scientific	75008150
Microcentrifuge	Eppendorf	5425R
Cell Incubator Steri-Cycle i250	Thermo Fisher Scientific	Forma Series II
Biosafety cabinet ninoSAFE class II	AB Nino Lab	N/A
1000 μL tips, filtered sterile wide bore	Thermo Fisher Scientific	2079GPG
Round cover glass #1.5, 8mm	Electron Microscopy Sciences	72296–08
Thin forceps, Inox 02, Style 4	Dumont	C102-4-PO
Eclipse Ti inverted microscope	Nikon	
Zyla sCMOS camera	Andor	VC-884
SOLA light engine	Lumencor	
Round cover glass, #1.5, 12mm	VWR	630–2190
Flow cytometer	BD Biosciences	BD LSRFortessa
Flow cytometer tubes	Corning	352008
UltraComp eBeads™ Compensation beads	Invitrogen	01-2222-41
Light/fluorescence microscope (cell culture)	Nikon	Ts2-FL
Fluorescence microscope (live calcium imaging)	Nikon	ECLIPSE FN1
16× DIC water dipping objective	Nikon	CF175 LWD 16X W
Magnification variable turret (1×/1.25×/1.5×/2×)	Nikon	FN-MT
sCMOS camera	Teledyne Photometrics	01-KINETIX-22MM-M-C
LED light source	CoolLED	pE-800
Filter cube (DAPI/FITC/TRITC/CY5)	Chroma	Cat. no: 89401
Peristaltic pump	Ismatec	ISM834C
Tygon tubing 1,14 mm	Ismatec	Tygon LMT-55
Tygon tubing 3,17 mm	Ismatec	Tygon R3607
Water bath	Clifton	NE1D-4
Drip chamber	CODAN	Infusion set L86
Temperature controller	Custom built in-house	N/A
Recording chamber	Custom built in-house	N/A
Fixation ring	Custom built in-house	N/A
Perfusion heater	Custom built in-house	N/A
Fine-tipped forceps	Sigma-Aldrich	N/A
Borosilicate glass bottle, 100 mL	VWR	215–1592
Sealing film	Amcor	Parafilm M
Bubbling stone for gas dispersion	N/A	N/A

## Materials and equipment

Various factors are needed in the media for microglia differentiation. These are Human Bone Morphogenic Protein-4 (BMP-4), recombinant human stem cell factor (SCF), recombinant human vascular endothelial growth factor (VEGF), recombinant human macrophage colony stimulating factor (M-CSF), recombinant human interleukin-3 (IL-3), recombinant human granulocyte-macrophage colony stimulating factor (GM-CSF), and recombinant human interleukin-34 (IL-34) and should be prepared beforehand, according to the table below.

**Table:** Factor preparation

Factor	BMP-4	SCF	VEGF	M-CSF	IL-3	GM-CSF	IL-34
Weight	10 μg	10 μg	10 μg	50 μg	10 μg	5 μg	50 μg
H^2^O	-	50 μL	20 μL	50 μL	400 μL	50 μL	50 μL
0.1% BSA in H_2_O	180 μL	450 μL	180 μL	450 μL	-	450 μL	450 μL
5 mM HCl	20 μL	-	-	-	-	-	-
**Final conc.**	50 μg/mL	20 μg/mL	50 μg/mL	100 μg/mL	25 μg/mL	10 μg/mL	100 μg/mL
Aliquot	10 μL	10 μL	10 μL	50 μL	50 μL	50 μL	50 μL
Storage temp	−20°C	−20°C	−20°C	−20°C	−20°C	−20°C	−20 °C
Storage time (up to)	3 months	12 months	12 months	12 months	3 months	12 months	3 months

**Table:** Embryonic Body Medium (EBM)

Reagent	Final concentration
mTesR+ medium	5 mL
Human Bone Morphogenic Protein-4 (BMP-4)	50 ng/mL
Recombinant human stem cell factor (SCF)	20 ng/mL
Recombinant human vascular endothelial growth factor (VEGF) 121	50 ng/mL
Rock inhibitor	1 μL/mL

Store at 4°C for up to 1 week.

**Table:** Hematopoietic medium (HM)

Reagent	Final concentration
X-Vivo 15	50 mL
Glutamax	500 μL (1×)
Pe/St 100×	500 μL (1×)
b-mercaptoethanol	55 μM
Recombinant human macrophage colony stimulating factor (M-CSF)	100 ng/mL
Recombinant human interleukin-3 (IL-3)	25 ng/mL

Store at 4°C for up to one week.

**Table:** Microglia Medium (MiM)

Reagent	Final concentration
Advanced DMEM/F12	50 mL
Glutamax	500 μL (1×)
Pe/St 100×	500 μL (1×)
b-mercaptoethanol	55 μM
Recombinant human granulocyte-macrophage colony stimulating factor (GM-CSF)	100 ng/mL
Recombinant human Interleukin-34	10 ng/mL

Store at 4°C for up to one week.

**Table:** Blocking Buffer

Reagent	Final concentration	Volume for 10 mL
TBS	N/A	9.47 mL
Triton X-100	0.3%	30 μL
Donkey serum	5%	500 μL

Prepare fresh and store at 4°C overnight.

**Table:** AAV transduction

Reagent	Final concentration
AAV particles (≥1 × 10^12^ vg/mL)^a^	1.5 × 10^8^–1.5 × 10^9^ vg/mL
Sterile PBS, pH 7.4 (pre-chilled to 4°C)	1×
Doxorubicin^b^	300 nM
Cell medium (MiM)	1×

^a^ Store long-term at −80°C ^b^ Store 2 mM stock at −20°C for up to 6 months.

**Table:** FACS buffer

Reagent	Final concentration	Amount
Fetal bovine serum	5%	2.5 mL
Sterile PBS, pH 7.4	1×	47.5 mL
**Total**	**N/A**	**50 mL**

Store at 4°C for up to one week.

**Table:** Dyes

Reagent	Final concentration	Ex/Em (nm)
NucBlue™	1 drop per 0.5 mL	360/460
LIVE/DEAD™ Fixable Far Red™	1:1000 (1 × 10^6^ cells/mL)	633/655

**Table:** ACSF

Reagent	Final concentration (1×)	Amount
NaCl	120 mM	7013 mg
NaH_2_PO_4_	1 mM	138 mg
NaHCO_3_	26 mM	2184 mg
KCl	2.8 mM	209 mg
D-Glucose	3.5 mM	630 mg
ddH_2_O	N/A	Add to 1000 mL
**Total**	**N/A**	**1000 mL**

Store at 4°C for up to 1 month.

**Table:** ATP stock solution

Reagent	Final concentration	Amount
ATP disodium salt	100 mM	551 mg
DMSO	N/A	10 mL
**Total**	**N/A**	**10 mL**

Aliquot and store at −20°C for up to 6 months.

**Table:** Magnesium stock solution

Reagent	Final concentration	Amount
MgCl_2_	200 mM	4066 mg
ddH_2_O	N/A	Add to 100 mL
**Total**	**N/A**	**100 mL**

Store at 4°C for up to 1 month.

**Table:** Calcium stock solution

Reagent	Final concentration	Amount
CaCl_2_	200 mM	2940 mg
ddH_2_O	N/A	Add to 100 mL
**Total**	**N/A**	**100 mL**

Store at 4°C for up to 1 month.

### Equipment

**Figure 1:**
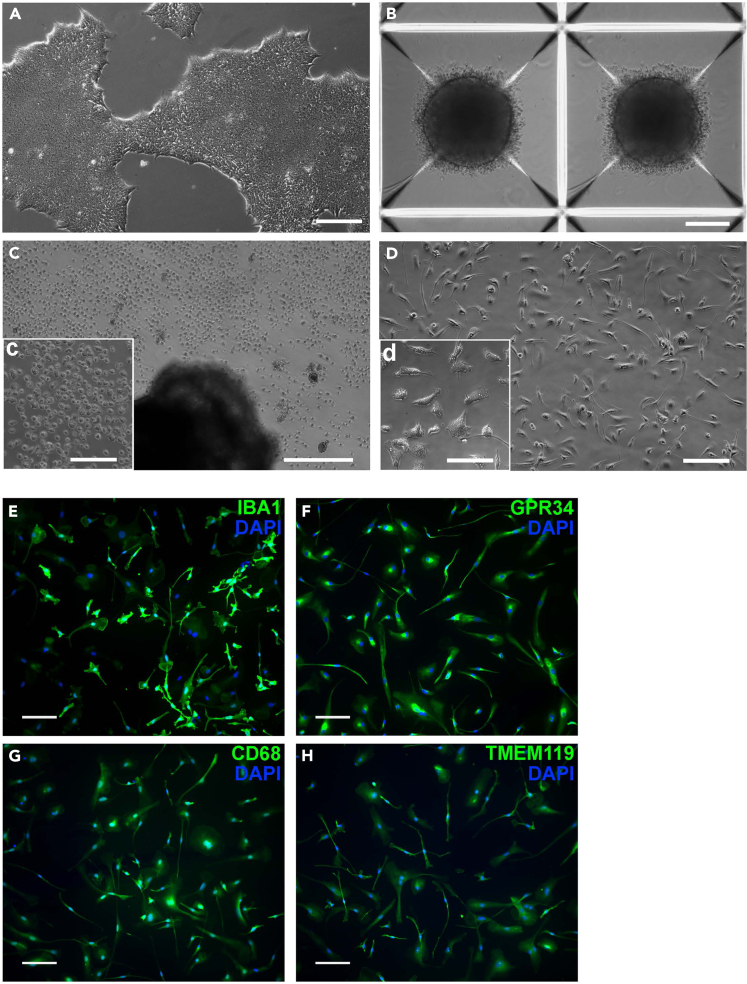
Differentiation of hiPSC-derived microglia (A–D) Light microscopy images: Images of (A) hiPSCs, (B) embryonic bodies in Aggrewells, (C) embryonic bodies producing progenitors (magnified image in C), and (D) microglia (hiMG) at differentiation day 5 (magnified image in D). Scalebars: 200 μm except for d (100 μm). (E–H): Fluorescent images: microglia stained for (A) IBA1 (1:500), (B) GPR34 (1:100), (C) CD68 (1:300), (D) TMEM119 (1:100), cell nuclei are stained with DAPI, scalebars: 100 μm.

For the images presented in Figures 1A–1D, a brightfield microscope was used. To image microglial markers after staining, a fluorescence microscope with suitable excitation/emission filters for the antibody-conjugated fluorophore(s) used is needed. Here, a Nikon TiEclipse inverted microscope with a Zyla Andor sCMOS Camera was used.

To record calcium dependent increases in fluorescence emission from GCaMP8s, the protocol uses a sCMOS camera connected to a microscope equipped with a 16× water dipping objective (with magnification turret), a 470 nm excitation light source, and a FITC filter cube to record calcium dependent increases in fluorescence emission from the genetically encoded sensor GCaMP8s. Live data is collected using NIS-Elements (other licensed or open-source software may be used) and analyzed using the free software Fiji.

***Note:*** The perfusion system, while highly customizable, should minimally include a peristaltic pump, tubing, a heat source for temperature control, and a gas delivery system. To improve physiological relevance in recordings, the temperature and composition of the extracellular solution may be adjusted to mimic human cerebrospinal fluid. A pH of 7.4 is maintained by bubbling the circulating ACSF with a mixture of O_2_ and CO_2_ (95% oxygen, 5% carbon dioxide).

***Note:*** Calcium imaging should be performed in a dark environment to maximize signal-to-noise ratio and the quality of recordings.

## Step-by-step method details

### Generation of hiPSC-derived microglial cells

**Timing: 1.5 months**

**Figure 2:**
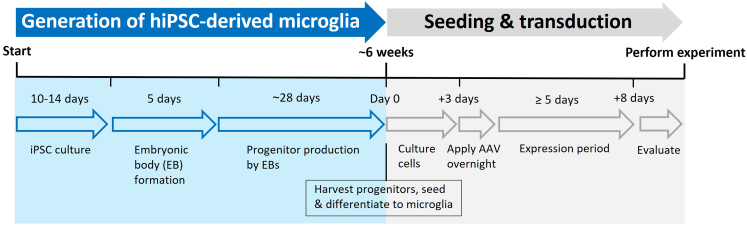
Timeline of key steps in the generation and viral transduction of hiMG cells

hiPSCs may either be acquired commercially or generated in-house. The cells are best expanded and stored in aliquots in liquid nitrogen to ensure reproducibility over a longer period. For the differentiation to hiMGs, one vial of hiPSCs is thawed, cells are grown and expanded to generate embryonic bodies, which then serve as microglia factories and produce microglial progenitor cells. The progenitor cells are harvested and seeded at the required density and differentiate into mature hiMGs within 5–7 days. The overall time to generate microglial cells from hiPSCs is about 6 weeks (see Figure 2) and continuous harvest of microglial progenitor cells is possible for up to several months.

***Note:*** All cells are grown and kept in an incubator set for 37°C and 5% CO_2_. All centrifugation steps are at room temperature (RT).

1. Coating with Matrigel:
  a. Prepare Matrigel after manufacturer’s instructions, or as published elsewhere,^11^ and store aliquots at −20°C.
  b. Thaw aliquot overnight in fridge. 15 μL of Matrigel are added to 1 mL cold DMEM per 6-well.
  c. Ensure the well-floor is evenly covered and incubate plate for 1h at RT or for 1–3 days in fridge.

***Note:*** Keep aliquot of Matrigel in cold block during these steps and return to fridge as soon as possible. Matrigel aliquots should be kept in fridge for up to one week and not be frozen again after thawing. To reduce the risk of contamination and to prevent drying, seal plate with parafilm when stored for more than 1 h.

2. Thawing and expansion of hiPSCs.
  a. Prepare mTesR^+^ medium by adding the supplement to the basal medium, prepare 45 mL aliquots, and freeze for longer storage at −20°C.
  b. Thaw one vial (aliquot) of hiPSCs in a 37°C water bath.
  c. Transfer cells into a 15 mL-tube and slowly add 10 mL pre-warmed mTesR^+^ media.
  d. Centrifuge at 200 *g* for 5 min.
  e. Discard supernatant and resuspend cell pellet in 2 mL mTesR^+^ medium.
  f. Remove Matrigel from well and transfer cell suspension evenly to well.
  g. Change media daily.
3. Passage and expansion of hiPSCs.
  a. Matrigel coating should be done beforehand as described above.
  b. When cells reach 80%–90% confluency, remove media and wash with sterile filtered DPBS^−/−^.
  c. Add 5 mM EDTA in PBS to cells (1 mL/6-well) and incubate for 3–5 min at 37°C.
    ***Note:*** Check cells after 3 min under microscope, when they start to round up it is time to proceed with the next steps.
  d. Remove EDTA and detach cells carefully with 1 mL mTesR^+^ by pipetting up and down, max 3 times. Repeat with new mTesR^+^ until most cells are collected.
  e. Seed out in newly coated wells, note down passage number and dilution (e.g., 1:4).
  f. Change media daily.
  g. After at least 1 week in culture, aim for a final passage into 3× 6-wells and grow until 80% confluency is reached (see Figure 1A). These cells are then used for the formation of embryonic bodies.
    ***Note:*** Passage number should be kept to a minimum and unnecessary long culture time should be avoided.
4. Embryonic Body formation.
  ***Note:*** The measures below are for 1 AggreWell 800, 24-well plate.
  a. Prepare Embryonic Body Medium (EBM).
  b. Remove the media of 3 wells with hiPSCs and wash with sterile filtered DPBS^−/−^.
  c. Add 1 mL TryPLE EXPRESS per well and incubate for 5 min at 37^°^C to detach cells.
  d. Pipette cells up and down to achieve a single-cell suspension.
  e. Collect cells in 2 mL mTesR^+^/well.
  f. Assess cell number and viability (typically >90%) from a 200 μL aliquot of the cell suspension and by using a cell counter (e.g., NucleoCounter® NC-200).
  g. Centrifuge 4 × 10^6^ cells at 200 *g* for 5 min.
  h. Discard supernatant and resuspend cell pellet in 1 mL EBM and keep at 37^°^C until further use.
  i. Add 500 μL of anti-adherence rinsing solution to 1 AggreWell™ 800 (24-well plate) and centrifuge the plate at 1300 *g* for 5 min.
    ***Note:*** Use swinging rotor suitable for plates as listed under “Machines and other equipment”.
  j. Remove anti-adherence rinsing solution and rinse well with 1 mL mTeSR^+^.
  k. Add 1 mL of EBM.
  l. Add 1 mL cell solution, carefully pipette up and down 2–3 times to mix, and immediately centrifuge at 100 *g* for 3 min.
  m. Change every day very carefully half the media (i.e., replace 1 mL with fresh EBM) without detaching the growing cell spheres (see Figure 1B).
  n. Prepare Hematopoietic medium (HM).
  o. After 4 days of daily half-media-change, on the fifth day resuspend EBs carefully by pipetting up and down twice using wide-bore 1000 μL tips.
    ***Note:*** Check under the microscope if there are remaining EBs, HM can be used to detach remaining EBs if needed.
  p. Transfer all EBs onto an inverted cell strainer 40–70 μm, placed onto a 50 mL tube.
  q. Place cell strainer correctly onto a new 50 mL tube and wash EBs from cell strainer with 6 mL HM.
  r. Distribute EBs equally in a full 6-well plate with 3 mL HM per well.
  s. Place the plate in the back of an incubator.
  t. Once a week 2 mL HM are exchanged and after about 3 weeks additional media is added every 3–4 days when the media color turns slightly orange.
    ***Note:*** It should be at least 3 mL in each 6-well due to potential evaporation.
    **CRITICAL:** Do not touch or move the plate between the first media changes!
5. Differentiation of microglial progenitor cells to hiMGs (see problems 1, 2, 3, and 5 for troubleshooting).
  ***Note:*** After about 4 weeks, clouds of progenitors can be seen floating in the well (Figure 1C).
  a. Prepare microglia medium (MiM).
  b. Harvest cells from at least two wells and gather in a tube.
  c. Take an aliquot and analyze cell count and viability (use e.g., a NucleuCounter® NC-200 and aim for >95% viability and <10% aggregates).
    ***Note:*** If viability is not high enough, wait 1 week and try again. If the percentage of aggregates is too high, gently pipette up and down a few times and repeat measurement.
  d. Seed cells at the required density (50.000 cells/cm^2^) in uncoated 96-, 48-, 24-, 12- or 6-well plates depending on the size and type of experiment.
    ***Note:*** The format used by us for microglia staining, AAV transduction and calcium imaging are found in respective protocol sections.
  e. Check under microscope to ensure even distribution of cells.
    ***Note:*** Gentle movement of the plate in an 8-shape helps to distribute cells evenly.
  f. After 1 h, check that cells have settled and exchange all media to MiM. Change the media every second day.
    ***Note:*** Even though microglia are not easily detached by pipetting, it is recommended to pipette gently over the wall to avoid damaging the cells with either the pipette itself or due to the pressure from pipetting.
    Cells will start to display microglia-like morphology (Figure 1D) already after 2–3 days but require differentiation until day 6 to mature and express typical microglial markers like IBA1 (Figure 1E), GPR34 (Figure 1F), CD68 (Figure 1G), and TMEM119 (Figure 1H).

### Microglial marker staining

**Timing: 3 days**

Microglia are harvested as described above, and cells are seeded onto sterile uncoated 8 mm coverslips in a 48-well plate (see problems 6, 7, and 8 for troubleshooting related to this staining protocol).

***Note:*** Coverslips can be placed in the wells by using sterile forceps. Coverslips are autoclaved prior to being used in cell culture to ensure sterility. Coverslips that are at least 3 mm less in diameter than well diameter enable easier removal with forceps after staining.

6. Fixation of hiMGs.
  a. At the end of the experiment, remove media and wash cells with sterile PBS.
  b. Remove PBS and add 200 μL Histofix/48-well for 12 min at room temperature (RT).
  c. Remove Histofix and add 200–300 μL PBS.

***Note:*** Cells can now be stored in fridge for 1–2 weeks before staining is initiated.

7. Staining of hiMGs for microglial markers.
  Prior to staining, prepare TBS-solution, which can be kept for several months at room temperature, and blocking buffer, which should be prepared fresh in the required amount for each staining and kept cool in between the steps.
  Day 1: Staining I
  a. Remove PBS and wash cells 3× for 5 min with 200 μL TBS.
  b. Remove TBS and add 200 μL blocking buffer for 1h at RT.
  c. Prepare primary antibody solution at the end of that incubation hour. Dilute the antibody in blocking buffer in the required dilution, e.g., IBA1 (host: rat) 1:500.
    ***Note:*** If a new antibody is used, the manufacturer’s suggested dilution is recommended, otherwise a tested dilution can be used.
  d. Remove blocking buffer and add 100 μL primary antibody solution per well. Incubate overnight at 4^°^C.
    Day 2: Staining II
  e. Remove primary antibody solution and wash 3× for 5 min with 200 μL TBS.
  f. During the washing steps, prepare secondary antibody solution with a suitable secondary antibody in blocking buffer (dilution 1:500), depending on the utilized primary antibody (e.g., if IBA1 (host: rat) was used, use donkey anti-rat IgG Alexa Fluor 488).
    ***Note:*** Always keep secondary antibody solution protected from light.
  g. Remove TBS, add 100 μL secondary antibody solution per well and incubate protected from light for 1 h at room temperature.
  h. Remove antibody solution and wash with 200 μL TBS for 5 min.
  i. Remove TBS and add 200 μL TBS with DAPI (1:1000) for 7 min.
    ***Note:*** DAPI should be prepared according to the manufacturer’s instructions.
  j. Remove DAPI-TBS and wash with 200 μL TBS for 5 min.
  k. Remove TBS and add 200 μL dH_2_O as a final washing step for 3 min.
  l. Add a 5 μL drop of ProLong gold onto a glass slide (where coverslip with cells is to be placed).
  m. Take up coverslip from well with thin forceps and place the side with cells downward, towards the ProLong Gold drop. Gently press down the coverslip to remove bubbles.
  n. Repeat steps l-m until all coverslips are mounted on glass slides.
  o. Store glass slides at room temperature protected from light for at least 24h. Long-term storage at 4^°^C is recommended.
    ***Note:*** Two to six 8 mm coverslips can be added beside each other on one glass slide. Store glass slides horizontally for the first 24 h.
    Day 3: Imaging
    Allow slides to dry for at least 24 h.
  p. Image cells with a fluorescent microscope.
    For the images presented in Figure 1E-H, a Nikon Ti Eclipse inverted fluorescence microscope was used together with an Andor Zyla sCMOS camera. Images were captured using the provided NIS software and a 20× air objective.

### AAV transduction

**Timing: 5**–**7 days**

***Note:*** Packaging and production of custom AAV particles using the cMG rep/cap plasmid may be performed in-house (see e.g., Negrini et al^12^), by a viral vector core facility or using a commercial service provider.

***Note:*** Cells should be seeded and cultured according to instructions in step 5, with media change every second day. We typically initiate the transduction protocol on day 3 after seeding (see Figure 2).

This part of the protocol describes key steps involved in delivery of transgenes to hiMGs using AAV-cMG (see problems 9 and 10 for troubleshooting). Solutions and reagents (see materials and equipment) can be prepared in advance.

#### Day 1

8. Prepare cells for transduction.
  a. Confirm good cell viability using a sterilized inverted cell culture microscope on the day of the experiment.
    ***Note:*** When examined with brightfield and phase-contrast microscopy, viable cultures typically feature evenly distributed and adherent cells, with thin and smooth edges and branched processes indicative of ramified or semi-ramified morphology. A mix of cells displaying branched and more amoeboid (round) morphology is normal. An abundance of small amoeboid cells with thick or jagged edges, or lots of floating cells, is a sign of low viability.
  b. Check that all solutions/reagents are ready for use and that laboratory hood is appropriately stocked before proceeding.
  c. Prepare an appropriate volume (depending on number and size of wells) of fresh MiM.
  d. Thaw a frozen aliquot of 2 mM doxorubicin stock to RT inside the hood (or use fresh).
    ***Note:*** Doxorubicin is used to enhance viral transduction/transgene expression. We recommend titration in the 100–500 nM range to determine optimum concentration (toxicity may be noticeable above 500 nM).
  e. Prepare a 15 mL Falcon tube containing cold sterile PBS and place on ice inside the hood.
  f. Thaw AAV vial(s) on ice next to PBS.
    ***Note:*** Handling of AAV should be performed in accordance with safety guidelines at the local institution.
  g. Prepare a pre-dilution (see note below) by adding sterile cold PBS (adjust for the total volume desired) to an empty Eppendorf tube and place on ice.
    ***Note:*** High-purity AAV particles are commonly pre-diluted in sterile cold PBS to achieve manageable working volumes, but virus can be added directly to the cell medium (Figure 3).

**Figure 3:**
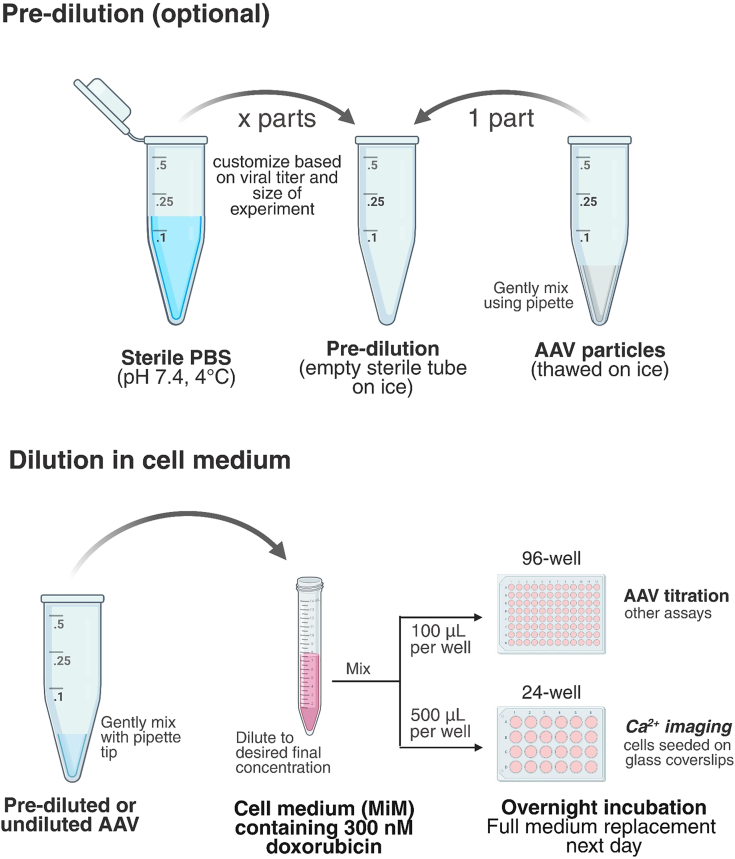
Stepwise overview of the transduction procedure Depending on viral titer, microplate format and number of wells to be transduced AAV can be pre-diluted or added straight to freshly made medium containing doxorubicin for subsequent overnight incubation.
9. Perform transduction.
  a. Carefully pipette up and down a few times to mix the contents of the AAV vial.
    ***Note:*** Take care not to introduce air bubbles.
    i. Add to the Eppendorf tube containing PBS on ice.
      ***Note:*** Adjust dilution factor and total volume according to viral titer and experimental format.
    ii. Aspirate/dispense a few times to make sure no AAV particles remain in the pipette tip.
  b. Add doxorubicin to freshly prepared MiM and dilute the virus to a desired final concentration.
    ***Note:*** Titration in the 1 × 10^8^–1 × 10^9^ vg/mL range is recommended for identification of optimal dose in this protocol. However, a larger titration span (e.g., from 1 × 10^5^10^10^ vg/mL) is suitable if starting from scratch.
  c. Fetch the cell plate from incubator and perform a full medium replacement using the AAV/medium mixture.
  d. Gently shake the plate in a north-south and then east-west direction and incubate with AAV overnight.

#### Day 2

10. Check cell viability and perform media change.
  a. In the morning, visually inspect cells under a sterilized inverted epi-fluorescence microscope and confirm good cell viability using brightfield microscopy (see step 1a).
    ***Note:*** A faint signal may be detectable upon excitation of the fluorescent protein marker at day 2.
  b. Prepare an appropriate volume of fresh MiM in a sterile Falcon tube.
    ***Note:*** From day 2 and onwards only standard MiM is used.
  c. Replace the full volume in each well with standard MiM.

#### Day 4

11. Repeat steps 9a-c. Perform a full medium replacement every second day from here on.

#### Days 5–7

**Figure 4:**
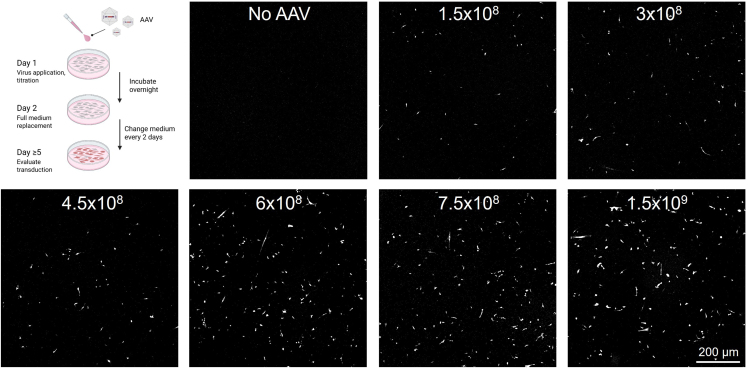
Overview of AAV titration experiment and expected results AAV particles were diluted to final concentration in cell medium containing 300 nM doxorubicin and applied (using a full medium replacement) to hiMG at indicated concentrations (vg/mL). Fluorescence imaging of mScarlet expression was performed in live cultures 5 days post transduction. Optimal concentration was found to be around 6–8 × 10^8^ vg/mL.

***Note:*** At this point, a large majority of cells should be fluorescent and display a similar range of morphologies as seen in un-transduced cultures. A DAPI or Hoechst stain can be used to quantify transduction efficiency after fixation (see microglia marker staining protocol).

12. Confirm successful transduction of microglia using a sterilized inverted epi-fluorescence microscope (cf. Figure 4).

13. Perform the desired experiment or proceed to section below.

### Quantification of transduction efficiency using flow cytometry

**Timing: 3 h**

**Figure 5:**
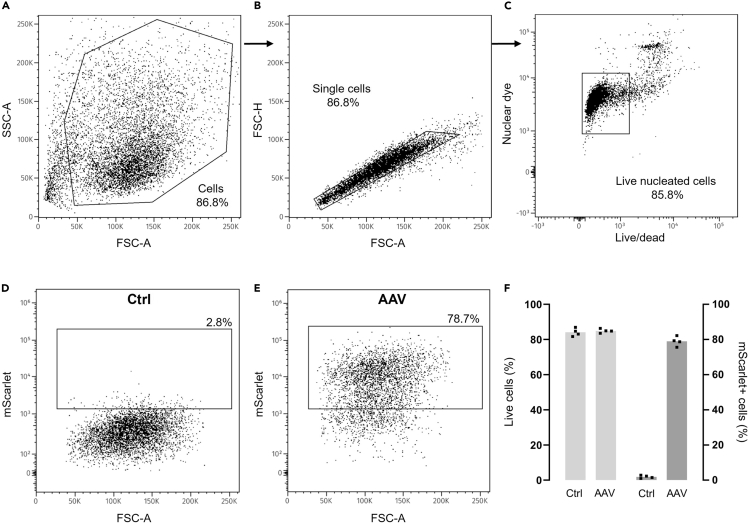
Expected results from flow cytometry analysis of viral transduction using mScarlet as marker (A) Analysis of total cell amount based on cellular size (forward scatter-area, FSC-A) and granularity (side scatter-area, SSC-A). (B) Analysis of single cells based on FSC-A versus forward scatter-height, FSC-H. (C) Analysis of live nucleated cells defined by viability dye (LIVE/DEAD) versus nuclear dye (NucBlue). (D) Representative dot plot showing the percentage of mScarlet-positive hiMG versus FSC-A in control sample (no AAV). (E) Representative dot plot showing the percentage of mScarlet-positive hiMG versus FSC-A 5 days post transduction. (F) Bar graph summary of transduction and cell viability data. Average values and individual replicates are shown (N = 4 replicates per group).

The following procedure allows for determining the percentage of transduced viable cells following incubation with AAV using flow cytometry (Figure 5). It is based on a 96-well plate format (readily scalable) and supports, in addition to cell viability and nuclear staining, analysis of GFP and mScarlet (or markers of choice with similar ex/em spectra).

14. Prepare cells for flow cytometry.
  a. Starting at 5 days post viral transduction, prepare single-cell suspensions from adherent hiMGs by washing cells with DPBS^−/−^.
    ***Note:*** Transgene expression will continue to increase beyond 5 days post transduction, which will impact transduction efficiency using this readout as more cells are expected to register above detection threshold. For most AAVs (not including self-complementary) the time for transgene expression to reach maximum level is typically 3–4 weeks, with some variation due to target cell type, AAV serotype, type/size of payload, etc.
  b. Add 50 μL 0.25% Trypsin-EDTA and incubate for 5–10 min at 37°C.
  c. Inactivate trypsin by adding a 3× volume (150 μL) of FACS buffer (PBS supplemented with FBS).
  d. Add LIVE/DEAD™ Fixable Far-Red dye (1:1000) and gently pipette mix in well to disperse cell aggregates.
    ***Note:*** Cells should be minimally exposed to light throughout the protocol. At this stage the cells may be fixated, immunostained and stored at 4°C until data acquisition in flow cytometer.
  e. Transfer suspensions to 1.5 mL Eppendorf tubes and centrifuge at 300 × *g* for 5 min at RT.
    ***Note:*** Replicates from individual wells may be pooled in a 1.5 mL tube to increase sample cell numbers using a 96-well format.
  f. Discard supernatant and add 1 drop NucBlue™ dye per 0.5 mL cell suspension.
  g. Resuspend cells in cold FACS buffer and store on ice. Incubate with dye for 10–15 min.
    ***Note:*** The nuclear dye will have time to penetrate the cells during the sample preparation period.
  h. Keep the samples on ice until ready for acquisition at the flow cytometer.
  i. Centrifuge samples (300 × *g*, 5 min at RT) to remove excess dye, resuspend in 0.5 mL cold FACS buffer and transfer to flow cytometry tubes. Keep the samples on ice.
15. Prepare the instrument.
  a. Prepare the BD LSRFortessa by calibrating and optimizing the voltages and acquisition parameters using BD FACSDiva™ software according to the instructions in the software reference manual. If necessary, consult responsible FACS technician.
  b. Prepare compensation controls using UltraComp eBeads™ Plus Compensation Beads according to manufacturer’s instructions or use cells for single color controls.
  c. Set up an acquisition worksheet in the BD FACSDiva™ software to enable visualization of sample acquisition. Select dot plots to use the following gating strategy:
    i. Define total cells based on cellular size (forward scatter-area, FSC-A) and granularity (side scatter-area, SSC-A).
    ii. Define single cells based on FSC-A versus forward scatter-height, (FSC-H).
    iii. Live and nucleated cells (viability dye-negative in APC vs. NucBlue™ in BV421).
    iv. Transduction marker mScarlet in PE vs. FSC-A.
16. Collect, store and analyze data.
  a. Run a test sample flow rate using a spare sample and adjust to achieve a flow rate of 1,000–5,000 events/second.
  b. Acquire and record data from all samples.
  c. Export the data as a.FSC file and import into the FlowJo™ software (BD Life Sciences).
  d. Use single color compensation controls to create and apply a compensation matrix. Apply the compensation matrix to all experimental samples.
17. Analyze the data in FlowJo™.

### Calcium imaging

**Timing: 1 day**

**Figure 6:**
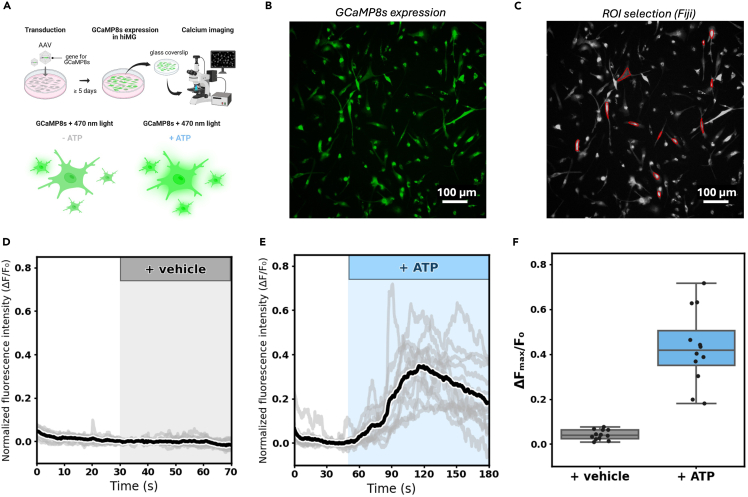
Calcium imaging using ATP stimulation in human iPSC-derived microglia expressing GCaMP8s (A) Schematic overview of the experiment (GCaMP8s-expressing hiMG areshown in green). (B) GCaMP8s expression (green cells) in a hiMG culture 5 days post transduction. (C) Region of interest (ROI) selection for 12 cells shown in red. (D-E) Normalized time-lapse fluorescence intensity data from ROIs in C and effect of vehicle (D) versus ATP (E) wash-in (as indicated). (F) Summary box-whisker plot showing maximum ΔF values from individual ROIs in the vehicle versus ATP stimulation group. Median values are shown as horizontal lines inside boxes.

This section of the protocol describes steps for live imaging of microglial calcium dynamics (Figure 6A) after seeding hiMG on 12 mm glass coverslips (see Microglia marker staining section) and transducing them (see Figure 3) with AAV-cMG encoding GCaMP8s. All solutions (see materials and equipment) can be prepared in advance, preferably on the day before the experiment.

**Figure 7:**
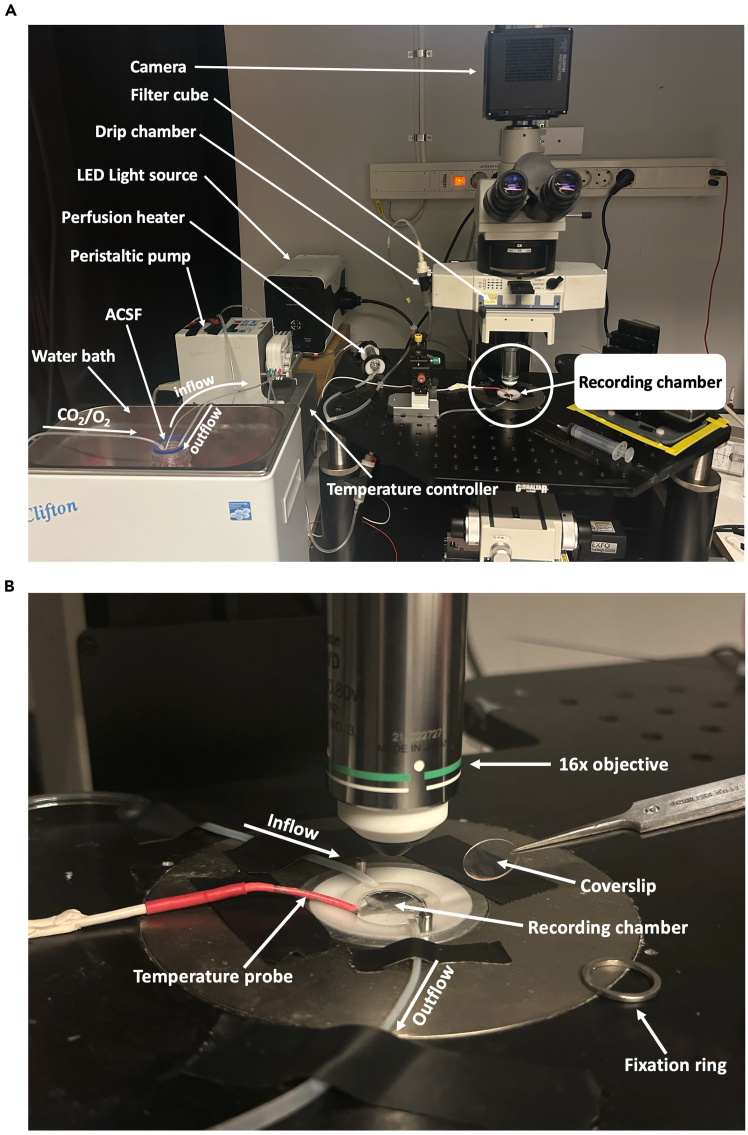
Equipment used for calcium imaging (A) Overview of the setup and key components. (B) Close-up showing the recording chamber setup.
  d. Allow the cells to acclimatize in the recording chamber for 10 min.
  e. Open NIS-Elements and confirm communication with hardware (camera, LED light source etc.).
  f. Go to live acquisition and confirm the expression of GCaMP8s in hiMG by turning on the 470 nm (blue) LED light. Cells expressing GCaMP8s will by default appear as white on a dark background (if desired this can be modified using pseudo-coloring options in NIS-Elements).
    ***Note:*** A brightfield (white) light source may be used to help initially identify and focus the objective on the cell layer prior to fluorescence illumination.
  g. Adjust the LED light to the lowest intensity that still allows for visualization of a clear fluorescent cell signature. Make sure all other light sources are turned off.
    ***Note:*** High-intensity LED illumination should be avoided (especially for prolonged periods) as it can cause cell heating and phototoxicity.
  h. Using the 470 nm LED, screen the glass for GCaMP8s-expressing viable cells and choose a suitable region of interest (ROI) (see Figure 8, problems 11 and 13).
    ***Note:*** The objective and magnification level used will determine the number of simultaneously recordable cells and image resolution.

18. Prepare solutions.
  a. Prepare the ACSF as described in materials and equipment.
  b. Prepare stock solutions of Mg^2+^ and Ca^2+^ as described in materials and equipment.
  c. Prepare the ATP stock solution.
19. Pre-imaging preparations.
  a. Measure 99 mL of pre-prepared 1× ACSF and transfer to a 100 mL glass bottle.
  b. Bubble the ACSF with 95% O_2_ and 5% CO2 to establish a pH of 7.4. Continue for the duration of the experiment.
  c. Add 550 μL of Mg^2+^ from stock solution directly to ACSF.
  d. Add 600 μL of Ca^2+^ from stock solution directly to ACSF.
    ***Note:*** Adding these volumes of calcium and magnesium to ACSF will produce physiological concentrations similar to those observed in healthy human CSF (Ca^2+^: 1.2 mM, Mg^2+^: 1.1 mM).
  e. Insert inflow and outflow tubes from the peristaltic pump to the ACSF.
  f. Turn on the peristaltic pump and circulate the ACSF at 1–3 mL/min to perfuse the recording chamber.
    ***Note:*** Use of a drip chamber may dampen pulsatile vibrations caused by the peristaltic pump. Maintaining a 0.5–1 cm column of ACSF is typically sufficient.
  g. Measure the temperature in the recording chamber and make sure it is stably maintained at 34°C.
    ***Note:*** In this protocol, a water bath and perfusion heater were used to maintain a recording temperature of 34°C. However, depending on perfusion setup and room temperature the need for this will vary.
  h. Cover the bottle opening with sealing film to minimize evaporation.
20. Prepare for calcium activity recording.
  a. Confirm that the perfusion is equilibrated in terms of temperature and flow rate and ensure that the recording chamber is free of mechanical vibrations and bubbles.
  b. Transfer a 12 mm coverslip containing GCaMP8s-transduced hiMG cells from the incubator to the recording chamber.
    **CRITICAL:** Perform a quick transfer of cells from the microplate well to recording chamber. If the incubator is not located near the imaging setup, you may transfer the coverslip to a transport plate together with its conditioned medium.
  c. Use fine-tipped forceps to gently place the coverslip in the recording chamber and secure it with the fixation ring (Figure 7). Pause the perfusion briefly during this step.

**Figure 8:**
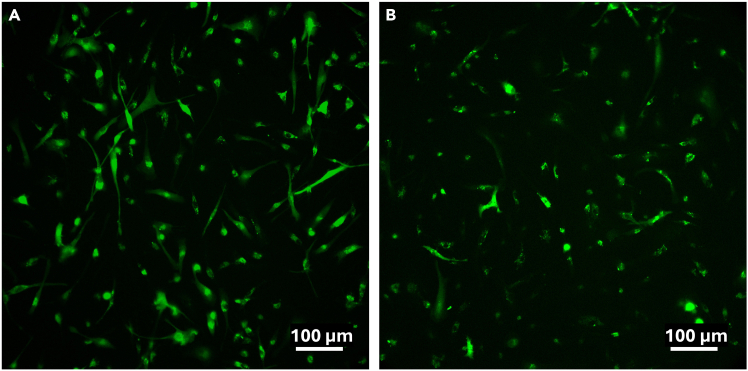
Comparison of two microglial cultures after transduction with GCaMP8s (A) Example of a culture suitable for calcium imaging. Viable cells with sensor expression throughout the cell body are plentiful. (B) Example of culture poorly suited for calcium imaging. Expressing cells are few and show less process extension.
  i. Turn off the LED light and leave the cells to rest for 5 min in dark.
21. Record calcium signaling in response to ATP stimulation (see Figures 6 and 9, Videos S1 and S2).
  a. Set up a time-lapse acquisition experiment of desired duration (e.g., 180 s) using a 5 or 10 Hz frame rate and choose a data storage location.
    ***Note:*** NIS-Elements will automatically save the imaging session as a.nd2 file to your desired location.
  b. Turn on the 470 nm excitation light (at pre-adjusted setting) and make sure that the ROI is still in focus.
  c. Press ‘Run now’ to start the acquisition.
    **CRITICAL:** Avoid light sources other than the 470 nm excitation light while recording and avoid touching the imaging setup during data acquisition.
  d. Add 100 μL of the ATP stock solution to the ACSF bottle (final concentration will be 100 μM).
    ***Note:*** Depending on perfusion setup and speed, the time it will take ATP to reach the recording chamber will vary. Estimate this time parameter and adjust the length of the recording session accordingly to capture a desired period of baseline activity and washout.
  e. Continue recording until the defined time window expires.
  f. Save the.nd2 file for later analysis in Fiji/ImageJ (or save as.tif stack for compatibility with other software of choice).
  g. Repeat steps 19–21 for desired number of coverslips.

**Figure 9:**
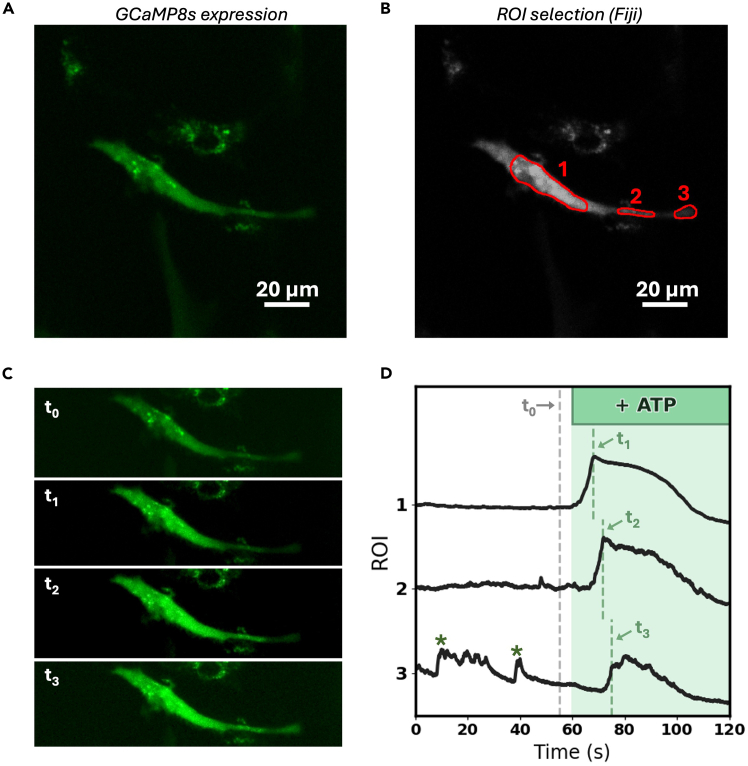
Propagation of calcium signal within microglia after ATP stimulation (A) Baseline GCaMP8s expression. (B) Regions of interest (ROIs): one somatic (ROI 1) and two distal regions (ROI 2 and 3) were chosen. (C) Snapshots showing propagation of the fluorescence signal within the cell following ATP stimulation. (D) Normalized fluorescence traces (ΔF/F0, F0 = mean fluorescence intensity over 10 s prior to stimuli) recorded from ROIs in B. The period shaded in green indicates when ATP was present in the recording chamber. ROI 3 (most distal) exhibits spontaneous activity prior to stimulation, indicated by asterisks. Dashed vertical lines indicate time points (t0-t3) corresponding to images in C.
  Methods video S1. ATP-induced calcium signaling and filipodia extension in hiMG, related to Expected Outcomes Time-lapse imaging (30× speed) of GCaMP8s fluorescence in hiMG, related to Figure 7.
  Methods video S2. Propagation of calcium signal in hiMG after ATP application, related to Expected Outcomes Time-lapse imaging (30× speed) of GCaMP8s fluorescence, related to Figure 8.

### Analysis

**Timing: 1**–**3 h**

Below we describe a simple protocol for ROI selection and fluorescence trace extraction using the open-source software Fiji/ImageJ. Note that this requires manual ROI selection and signal extraction. If a semi-automated approach is preferred, consider toolboxes such as CaImAn.^13^

22. Start Fiji.
23. Open the saved.nd2 file.
  a. ‘’**File**’’ -> ‘**’Open…**’’.
  b. Select your file.
24. Set measurements.
  a. ‘’Analyze’’ -> ‘’Set measurements…’’.
  b. Select ‘’**Mean gray value**’’.

***Optional:*** Optimize signal to noise with the options under ‘’**Image**’’, e.g. ‘’**Adjust**’’ -> ‘’Brightness/Contrast…’’.

***Optional:*** Fiji offers multiple tools for background subtraction, use if needed.

**Figure 10:**
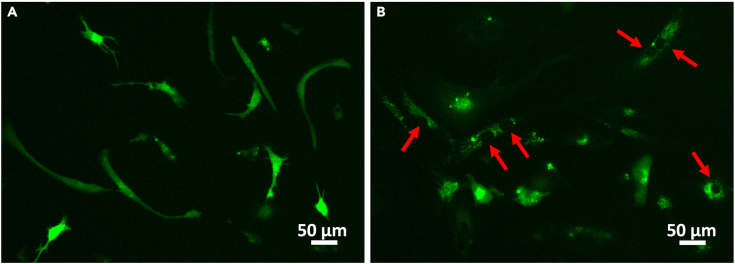
Comparison of GCaMP8s-expressing cells versus autofluorescent cells (A) Example of cells suitable for calcium imaging analysis. The fluorescence signal is homogenously distributed within the cell. (B) Examples of cells lacking sensor expression and displaying autofluorescence in response to 470 nm excitation (red arrows). The fluorescence signal is granular and typically localized to the perinuclear region. These cells will not report calcium signals.
  c. Press ‘**’Add [t]**’’ to save ROI.
  d. Repeat steps b-c for desired number of ROIs.
    ***Optional:*** Draw entire cells as ROIs or select multiple ROIs within the same cell for propagation analysis.
26. Measure fluorescence intensity.
  a. From ROI Manager ‘**’More >>**’’ -> ‘**’Multi Measure…**’’.
  b. Select ‘’Measure all X slices’’ and ‘’One row per slice’’.
  c. ‘’**Ok**’’.

***Note:*** If the mean gray value for an ROI is only measured for one slice despite selecting ‘’Measure all X slices’’, the ROI was applied only to that specific slice when selected (see problem 14).

25. Perform ROI selection.
  a. ‘’**Analyze**’’ -> ‘’**Tools**’’ -> ‘’**ROI Manager…**’’.
  b. Manually select ROI with the ‘’**Freehand selections**’’ tool.
    ***Note:*** Avoid selecting autofluorescent cells (see Figure 10). In case of difficulties choosing suitable ROIs, refer to the troubleshooting section (Problem 13).

27. Export the data.
  a. ‘’**File**’’ -> ‘’**Save as…**’’.
    ***Note:*** The default file format is.csv. If Microsoft Excel is preferred for analysis, do not change this. In this protocol, the file is saved as.txt for improved compatibility with Visual Studio Code and Jupyter-based data analysis. However, alternative formats or software may be used.
  b. ‘’**Ok**’’.
28. Analyze the data using a software of choice.

## Expected outcomes

In this protocol we describe step-by-step instructions for generation of human iPSC-derived microglia, efficient AAV transduction, and expression of a genetically encoded sensor for fluorescence imaging of calcium dynamics in these cells.

After successful differentiation, hiMGs should display a characteristic ramified or hypertrophic morphology with active process motility and phagocytic capacity.^2^ They should further express microglial markers (e.g., IBA1, CX3CR1, P2RY12, and TMEM119) and react to external stimuli, for example by increased proinflammatory gene expression upon stimulation with bacterial proteins.^1^^,^^2^ Cells are expected to be viable for up to 14 days, potentially longer, when maintained with appropriate media changes and seeding density.

Using the described vectors and transduction protocol the user can expect to achieve a transduction efficiency of near 80%, a viability similar to that of non-transduced controls (>80%) and high reproducibility (Figure 5). Results can be expected to vary depending on the time period allowed for gene expression after transduction, choice of promoter and gene(s) of interest, as well as the length of time spent preparing cells (and their handling) for analysis.

Following expression of GCaMP8s in hiMGs the distribution and propagation of spontaneous and evoked calcium signals, acutely and over repeated sessions, may be studied in response to a variety of experimental manipulations in multiple cells simultaneously. Variability in the number of GCaMP8s-positive cells is expected depending on general parameters of AAV transduction and composition (see above). Moreover, the quality and viability of hiMGs prior to, and throughout, the GCaMP8s expression period is a key determinant of successful data acquisition. If these conditions are met the user can expect to acquire time-lapse fluorescence data at high signal-to-noise (Figures 6 and 9, Videos S1 and S2) for analysis of temporal and spatial parameters of interest.

## Limitations

Although this protocol allows for the generation of hiMGs, the cells remain in an artificial *in vitro* setting, which cannot capture the complexity of a brain environment. This model allows therefore only for the analysis of hiMGs in monoculture environment, but does not model interactions with neurons, astrocytes, or peripheral immune cues, which can influence microglia behavior in vivo.

Differentiation efficiency might vary between cell lines and batches and might therefore require primary testing of hiMGs from different iPSC lines before experiments are initiated.

A limitation of the transduction protocol is that it focuses on hiMGs in monocultures. As no capsid variant exists that selectively infects microglia, the AAVs used in this protocol (including capsid and genome) would likely generate transgene expression in other cell types if used in mixed iPSC cultures/organoids. Thus, for such applications, the use of specific promoters and miRNA target sequences are recommended to limit expression to hiMGs.^14^ Furthermore, we cannot guarantee that similar results will be obtained using hiMGs generated using other human iPSC lines or differentiation protocols. In addition, a well-known shortcoming of all AAV variants is the payload capacity of 4.5 kb, limiting the number and size of GOIs that can be delivered.

Due to its calcium binding ability, the GCaMP8s molecule not only functions as an optical sensor but also acts as an intracellular calcium chelator. This property can itself affect calcium-dependent signaling processes, and ultimately the function of the studied cell,^15^ and constitutes a limitation of this approach. A further limitation is that *in vitro* culturing conditions poorly mimic the physiological brain environment and complexity that is known to shape microglial calcium signaling in the intact brain.^9^ Moreover, although hiMG share many properties with their *in vivo* counterparts, differences e.g., in parameters such as membrane receptor expression/distribution and intracellular calcium buffering could have significant impact on calcium-dependent signaling processes. Data acquired from calcium imaging in hiPSC-derived microglia should be interpreted considering these limitations.

## Troubleshooting

### Problem 1

Low cell concentration when collecting progenitor cells (related to generation of hiPSC-derived microglial cells step 5: Differentiation of microglial progenitor cells to hiMGs).

The cell yield can vary between hiPSC lines, differentiation age, and collection frequency. We typically collect sufficient numbers up to 3–4 months after starting differentiation.

### Potential solution

• Collect cells and determine total count (e.g., 100.000 cells/mL in 10 mL).
• Centrifuge at 400 × *g* for 5 min at RT.
• Discard supernatant.
• Resuspend cell pellet in MiM to reach the desired concentration (e.g., 4 mL to achieve 250.000 cells/mL).
• Count cells to get precise concentration and seed out at required cell density.

### Problem 2

Cells form clusters or show very high density in the center of the well (related to generation of hiPSC-derived microglial cells step 5: Differentiation of microglial progenitor cells to hiMGs).

When microglia are seeded out at too high density, cytokine availability (IL-34/GM-CSF) may become limiting. This can lead to cell death and surviving microglia migrate toward and cluster around cell debris. This can also happen when cells are seeded too closely to each other in e.g., the center of the well.

### Potential solution

• Seed cells at lower initial density.
• Ensure even distribution of cells before attachment by gently moving the plate.

### Problem 3

Harvested cells are present in clusters already at seed out (related to generation of hiPSC-derived microglial cells step 5: Differentiation of microglial progenitor cells to hiMGs).

Progenitors that are in culture for a longer time might attach to the well surface or form clusters with other progenitors.

### Potential solution

• Harvest only floating cells and avoid scraping or aspirating cells that are firmly attached to the well surface.
• Pipette harvested cells gently up and down to break up aggregates before counting.
• Do not seed cells when aggregates make up more than 10%; additional pipetting and re-counting is recommended.
• After seeding out cells, move plate gently in an eight-shape to ensure cells distribute evenly in the well. Check for even distribution under the microscope.

### Problem 4

Cells have amoeboid (round) morphology and/or detach and die (related to generation of hiPSC-derived microglial cells step 5: Differentiation of microglial progenitor cells to hiMGs).

When microglia are displaying a rounded amoeboid morphology and especially when they detach, this can have different causes.

• cells are lacking cytokines (IL-34/GM-CSF).
• cells are stressed.
• cells are too old.

### Potential solution

• Seed cells at lower initial density to ensure sufficient cytokine availability.
• Ensure media is prepared correctly:
  ◦ Ensure right cytokine concentrations.
  ◦ Prepare media with freshly thawed cytokines, avoid repeated freeze-thaw cycles.
  ◦ Use only media that is less than one week old.
  ◦ Store media in fridge, but warm it up to room temperature before adding to cells.
• Minimize mechanical stress by harsh pipetting or when carrying the plate.
• Minimize time outside the incubator or keep plates warm if cells are outside the incubator for a longer time.

### Problem 5

No microglia differentiation, cells remain fibroblast like (related to generation of hiPSC-derived microglial cells step 3: Passage and expansion of hiPSCs, and step 5: Differentiation of microglial progenitor cells to hiMGs).

iPSCs need to be fully pluripotent and exposed to correct cytokines at the right stage to differentiate towards microglia.

### Potential solution

• Verify iPSCs appear as shown in Figure 1A, ensure iPSC quality and confirm pluripotency by assessing markers such as OCT4, SOX2, or NANOG.
• Exclude fibroblast contamination by checking for fibroblast markers such as VIM, COL1A1, or FSP1.
• Extend maturation period.
• Test a new batch of cytokines.

### Problem 6

Microglia grow on both sides of coverslip (related to generation of hiPSC-derived microglial cells step 5: Differentiation of microglial progenitor cells to hiMGs, and microglial marker staining).

Microglia will grow after seedout in the well. Due to gravity, they will sink down to the well floor. However, if there is a gap between coverslip and well floor caused for example by a bubble, microglia will also infiltrate that space and might grow on both sides of the coverslip. It can often be observed that microglia grow to a certain extent on both side of the coverslip’s edges.

### Potential solution

• After adding the coverslip into the well, add 100 μL HM media and press the coverslip down gently. Ensure that no bubbles visible to the eye are left.
• Allow coverslip to settle before proceeding with adding microglia.
• Then add microglia by pipetting in a spiral starting in the middle of the well.
• Afterwards, press the coverslip gently down again, to ensure it remains pressed against the well floor.

### Problem 7

There is a lot of background signal (related to microglial marker staining).

### Potential solution

• Ensure you are not oversaturating the signal. Use a positive control or the well from your experiment with the expected strongest signal to ensure to capture the signal with the right exposure time, gain, or laser power.
• Check for autofluorescence of the sample using an unstained control.
• Ensure you perform the blocking step correctly.
• Ensure you do not let the cells dry during the staining process.
• Ensure you use the right concentrations of primary and secondary antibodies.
• Ensure you apply enough washing steps.
• Ensure the correct filter set or detection channel is used for the fluorophore.

### Problem 8

There is no signal after staining (related to microglial marker staining).

### Potential solution

• Ensure the light source of the microscope is working and that it can capture light in the wavelength of the secondary antibody.
• Ensure the correct filter set or detection channel is used for the fluorophore.
• Ensure you added the right concentration of primary and secondary antibodies.
• Ensure you added a suitable secondary antibody to the primary antibody.
• Include a positive control known to express the target protein.

### Problem 9

Low transduction efficiency (related to AAV transduction and quantification of transduction efficiency using flow cytometry).

In addition to AAV capsid variant the efficiency of hiMG transduction, when evaluated using a genetically encoded fluorescent reporter, is influenced by several parameters related to viral genome composition (choice of promoter, size and type of reporter, use of bi/multicistronic elements), delivery (concentration, incubation time, expression period) and handling (storage time and condition, time from thawing to incubation).

### Potential solution

• For AAV vector confirm:
  ◦ Original titer and final concentration.
  ◦ Proper storage conditions and handling. Avoid repeated thawing.
  ◦ AAV plasmid sequence.
• Confirm if doxorubicin was added and final concentration.
• Confirm that a minimum 5-day expression period was allowed.
• Check if non-transduced control cells are viable.

### Problem 10

Toxicity observed after AAV transduction (related to AAV transduction and quantification of transduction efficiency using flow cytometry).

AAV-dependent toxicity can occur as a result of capsid protein/DNA detection by the target cell (and subsequent anti-viral response), or from aggregation/misfolding of over-expressed reporters or other transgenes, and may be minimized by optimizing parameters of viral particle exposure (e.g., concentration, duration of incubation). Signs of AAV toxicity may include an abundance of small amoeboid cells, with thick or jagged edges, or lots of floating cells.

### Potential solution

• Confirm that intended final concentration of AAV was used.
• Confirm AAV purity and titer.
• Confirm doxorubicin dose.
• Exclude other sources of toxicity/cell death (e.g., contamination, incomplete medium composition, disrupted CO2 supply etc) by including non-transduced hiMG control.

### Problem 11

No visible GCaMP8s fluorescence in hiMG (related to calcium imaging section).

### Potential solution

See problem 9–10 to first exclude transduction-related issues as cause of this problem. Further confirm that:

• Cells are present and viable (check using regular light).
• LED light source is ON.
• Correct excitation wavelength, sufficient intensity, and exposure time is used.
• A compatible filter cube is used (e.g., FITC/GFP).
• Camera/software is in Live/acquisition mode (while excitation light is being provided).

### Problem 12

Cells show no or limited spontaneous/ATP-induced calcium signaling (related to calcium imaging section).

### Potential solution

• Confirm good viability of GCaMP8s-expressing cells (cf. Figure 8).
• Confirm that the ACSF (or other extracellular fluid) used contains at least 1 mmol/L of calcium. Check that pH and osmolarity of the solution is within physiological range.
• If no increase in fluorescence is seen with ATP, try using a higher concentration. Confirm that ATP is freshly prepared and properly dissolved. The frequency of spontaneous calcium events may vary as a result of ACSF composition/nutrient supply, recording temperature, cell density etc.).

### Problem 13

How to identify suitable/unsuitable ROIs for analysis (related to calcium imaging section)?

### Potential solution

Try to avoid selecting auto fluorescent cells as ROIs as they lack GCaMP8s expression. These cells typically display a granular fluorescence pattern in perinuclear areas (see example in Figure 8B) due to the accumulation of auto fluorescent material in organelles such as lysosomes.^16^^,^^17^ Circumvent this problem by selecting ROIs only from cells that display an even distribution of fluorescence throughout soma and processes (see examples in Figure 8A).

### Problem 14

ROI selection is limited to one frame (image) when measuring mean gray value in Fiji (related to calcium imaging section).

Sometimes a problem occurs where Fiji only allows ROIs to be stored on the frame where they were originally marked. As a result, the mean gray value will only be extracted from one frame rather than the entire image stack.

### Potential solution

Select the ROIs in the ROI Manager and go to ‘**’Properties…**’’. If ‘’**Position**’’ is specified (e.g., 125), change it to 0. This will apply the ROI to all frames.

## Resource availability

### Lead contact

Further information and requests for resources and reagents should be directed to and will be fulfilled by the lead contact, Andreas Björefeldt (andreas.bjorefeldt@gu.se).

### Technical contact

Technical questions on executing this protocol should be directed to and will be answered by the technical contacts, Stefanie Fruhwürth (stefanie.fruhwurth@gu.se) for iPSCs and hiMG and Andreas Björefeldt (andreas.bjorefeldt@gu.se) for AAV/calcium imaging.

### Materials availability

Plasmid sequences are available on request.

### Data and code availability

This study generated raw fluorescence data from calcium imaging using Fiji. Basic Jupyter-based scripts were created in Visual Studio Code with assistance from GitHub Copilot for data visualization and analysis. The data and scripts have not been deposited in a public repository but are available from the lead contact upon request.

## Acknowledgments

This study was supported by the Olle Engkvist Foundation (S.F.: 220-0223), the 10.13039/100007435Åke Wiberg Foundation (S.F.: M24-0212 and A.B.: M24-0026), Wilhelm & Martina Lundgrens Scientific Fund (S.F.: 2025-SA-4840 and A.B.: 2025-SA-4984), the Rune och Ulla Amlöv Foundation (S.F.: 2025-470), the Swedish Alzheimer’s Foundation (A.B.: AF-993056), the 10.13039/501100004722Lars Hierta Memorial Foundation (FO2023-0187 to C.N.H.), and the Tore Nilson Foundation (to C.N.H.).

## Author contributions

C.N.H. performed the work on hiPSC-derived microglia differentiation and immunostaining. A.M. performed the calcium imaging experiments and the associated data analysis. S.F. and A.B. assisted in experimental design and data interpretation and funded the work. All authors took part in preparation of the manuscript.

## Declaration of interests

The authors declare no competing interests.
